# Ultrasound is highly specific in diagnosing compensated cirrhosis in chronic hepatitis C patients in real world clinical practice

**DOI:** 10.1097/MD.0000000000016270

**Published:** 2019-07-05

**Authors:** Yi-Hao Yen, Fang-Ying Kuo, Chien-Hung Chen, Tsung-Hui Hu, Sheng-Nan Lu, Jing-Houng Wang, Chao-Hung Hung

**Affiliations:** a Division of Hepatogastroenterology, Department of Internal Medicine; b Department of Pathology, Kaohsiung Chang Gung Memorial Hospital and Chang Gung University College of Medicine, Kaohsiung, Taiwan.

**Keywords:** chronic hepatitis C, compensated cirrhosis, ultrasound

## Abstract

Ultrasound is routinely used during the evaluation of liver cirrhosis. Inter-observer variability is considered a major drawback. This retrospective study investigated the accuracy of ultrasound in diagnosing compensated cirrhosis (i.e., modified Knodell F3, F4) in chronic hepatitis C (CHC) patients in real world clinical practice. Consecutive treatment-naive CHC patients who underwent liver biopsy (LB) prior to interferon therapy from 1997 to 2010 were enrolled. Ultrasound was performed by 30 hepatologists prior to LB. Ultrasound-identified cirrhosis was defined as small liver size, nodular liver surface and coarse liver parenchyma. LB was used as a reference, and the diagnostic accuracy of ultrasound was assessed and compared. Fibrosis was scored according to the modified Knodell classification. A cohort comprising 1738 patients, including 922 men and 816 women with a mean age of 52.5 years, was analyzed in the present study. The distribution of the patients’ modified Knodell scores was F0 = 336, F1 = 489, F2 = 165, F3 = 315, F4 = 433. Ultrasound-identified cirrhosis was noted in 283 patients. Using ultrasound-identified cirrhosis to predict compensated cirrhosis, the sensitivity was 34.0%, the specificity was 97.1%, the positive predictive value was 89.8%, the negative predictive value was 66.1%, the positive likelihood ratio was 11.6, and the negative likelihood ratio was 0.68. The area under the ROC curve (AUROC) was 0.66.

Despite being affected by inter-observer variability, ultrasound is highly specific in diagnosing compensated cirrhosis in CHC patients in real world clinical practice. However, the sensitivity is low.

## 1 Introduction

Hepatitis C virus (HCV) infection is a major cause of chronic liver disease, with approximately 71 million chronically infected individuals worldwide.^[1,2,]^ The liver injury can range from minimal histological changes to severe fibrosis and cirrhosis with or without hepatocellular carcinoma (HCC).^[3]^ Clinical care for chronic hepatitis C patients has advanced considerably because of developments of direct antiviral agents (DAAs).^[3]^

According to current European Association for the Study of the Liver (EASL) guideline recommendation, assessment of liver fibrosis is necessary prior to antiviral therapy. Identifying patients with cirrhosis or bridging fibrosis is of particular importance, as their treatment regimen must be adjusted and post-treatment surveillance for HCC is mandatory. Fibrosis stage must be assessed by non-invasive methods initially, with liver biopsy reserved for cases where there is uncertainty or potential additional aetiologies.^[3]^ Transient elastography (TE) can be considered the non-invasive standard for the measurement of liver fibrosis.^[4]^

According to Baveno VI Consensus Workshop, the term “compensated cirrhosis” has been proposed to better reflect that the spectrum of bridging fibrosis and cirrhosis is a continuum in asymptomatic patients, and that distinguishing between the two is often not possible on clinical grounds.^[5]^

Ultrasound is routinely used during the evaluation of cirrhosis. In one prospective study of ultrasound in patients suspected of having cirrhosis who underwent liver biopsy, ultrasound had a sensitivity of 91% and a specificity of 94% for making the diagnosis.^[6]^ As for its limitations, inter-observer variability is considered a major drawback.

Until now, limited studies have reported the diagnostic accuracy of ultrasound in real world clinical practice. In this study, we sought to investigate the accuracy of ultrasound in diagnosing compensated cirrhosis (i.e., fibrosis with a modified Knodell score of F3, F4) in chronic hepatitis C (CHC) patients in real world clinical practice.

## 2 Patients and methods

### 2.1 Patients

**Figure 1:**
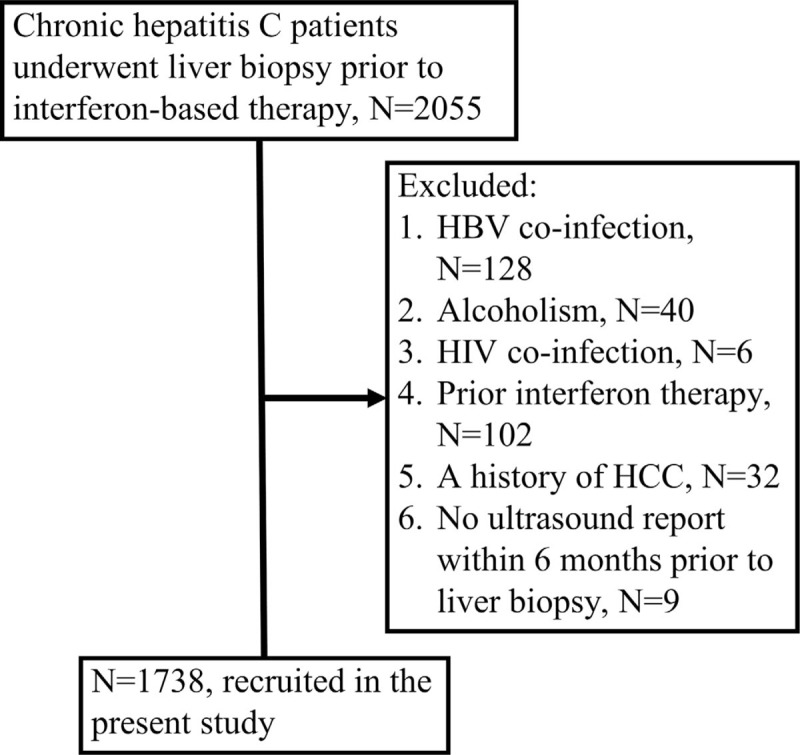
The inclusion and exclusion of subjects for this study.

This retrospective cohort study included consecutive adult CHC patients who had undergone liver biopsy prior to interferon and ribavirin therapy at Kaohsiung Chang Gung Memorial Hospital from December 1997 to October 2010. The diagnosis of CHC is based on the detection of both anti-HCV antibodies and HCV RNA.^[3]^ Patients with the following conditions were excluded from the study: the presence of chronic hepatitis B co-infection, human immunodeficiency virus (HIV) co-infection, alcoholism, HCC, prior interferon-based therapy, or no ultrasound reports within 6 months prior to liver biopsy (Fig. 1).

All the procedures used in the study were in accordance with the ethical standards of the responsible committees on human experimentation (institutional and national) and with the Helsinki Declaration of 1975, as revised in 2008. This study was approved by the Institutional Review Board of Kaohsiung Chang Gung Memorial Hospital (IRB number: 201601607B0). The requirement for informed consent was waived by the IRB. The data were analyzed anonymously.

### 2.2 Demographic and laboratory data

Demographic and laboratory data about these patients was reviewed by 1 investigator (YHY) to assess eligibility for the study. Ultrasound reports within 6 months prior to biopsy were reviewed.

### 2.3 Ultrasound

**Figure 2:**
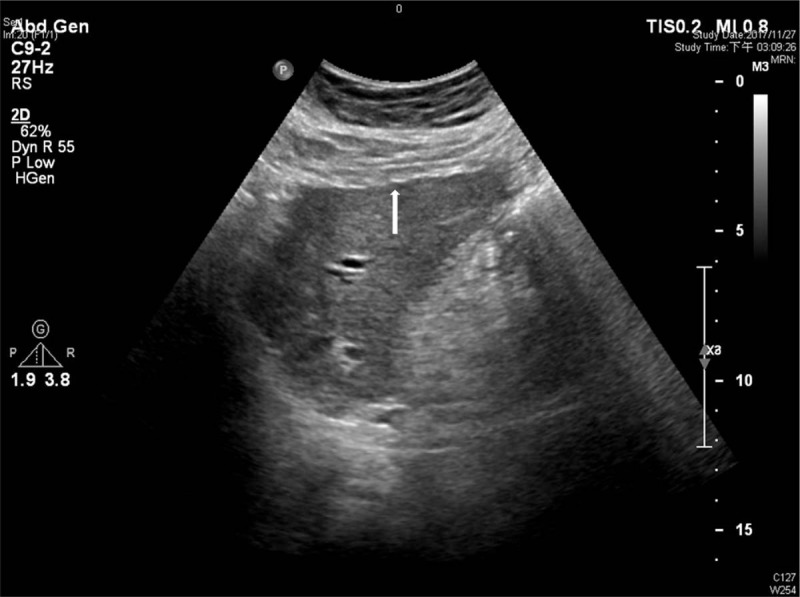
Example of nodular liver surface: Longitudinal view of the left lobe liver, liver surface appears as a dotted or irregular line *(arrow)*.

**Figure 3:**
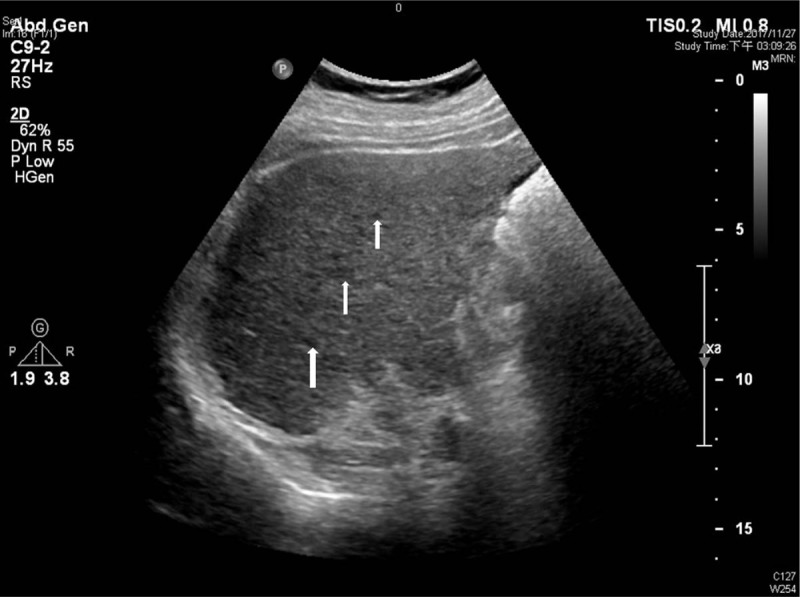
Example of coarse liver parenchyma: intercostal view of the right lobe liver, liver parenchyma shows areas of different echogenicity *(arrows)*, reflecting underlying nodularity.

Ultrasound was performed by 30 hepatologists in our department with 5 different ultrasound machines, including 2 Toshiba machines (an SSA-340 and an SSA-370; Toshiba, Tokyo, Japan), 2 Aloka machines (an SSD-680 and an SSD-2000; Aloka, Tokyo, Japan), and an HDI 5000 machine (ATL Ultrasound, Bothell). A diagnosis of cirrhosis was made with ultrasound when the liver appeared to be small and was accompanied by a nodular liver surface (Fig. 2) and coarse liver parenchyma (Fig. 3).^[7]^ Spleen size was calculated as the product of the oblique and diagonal diameters from spleen hilum and ≥20 cm^2^ was defined as splenomegaly.^[7]^

### 2.4 Liver histology evaluation

Each patient received an echo-guided percutaneous liver biopsy from the right hepatic lobe by using a 16-gauge Bard Max-core biopsy instrument. The sampling tissues were stained with H&E and reticulin silver. The degree of liver necroinflammation was calculated by Histology Activity Index scores.^[8]^ The degree of liver fibrosis was staged by modified Knodell histology index.^[9]^ Histology was reported by 3 pathologists, all of whom had no knowledge of the clinical characteristics of the study subjects. Compensated cirrhosis was defined as bridging fibrosis and cirrhosis, that is, modified Knodell score of 3 or 4.^[5]^

### 2.5 Statistical analysis

The continuous variables were summarized as appropriate in terms of mean and standard deviation or median and interquartile range, and the categorical variables were summarized in terms of frequency and percentage. The categorical variables were compared by chi-squared or Fisher exact tests, whereas the continuous variables were compared with the Student's *t* test. The diagnostic accuracy of ultrasound was assessed by the area under the receiver operating characteristic curves using liver biopsy as a reference. The area under the ROC curve (AUROC), sensitivity, specificity, positive predictive value (PPV), negative predictive value (NPV), and likelihood ratio for ultrasound examination were computed. All statistical analyses were performed by STATA version 11.0.

## 3 Results

### 3.1 Characteristics of the patients

The inclusion and exclusion of potential subjects for this study are depicted in Figure 1. Among 2055 screened CHC patients, 1738 (84.6%) were subsequently included. The characteristics of the patients are shown in Table 1. The mean age was 52.5 years, 53.1% of the patients were male, 46.7% of the patients were genotype 1, 48.6% of the patients were genotype 2, the mean necroinflammation score was 7.3, the median aspartate aminotransferase (AST) level was 91 IU/L, the median alanine-aminotransferase (ALT) level was 133 IU/L, 315 (18.12%) of the patients had a fibrosis score of 3, 433 (24.91%) of the patients had cirrhosis, and 283 (16.28%) of the patients had ultrasound-identified cirrhosis.

**Table 1:**
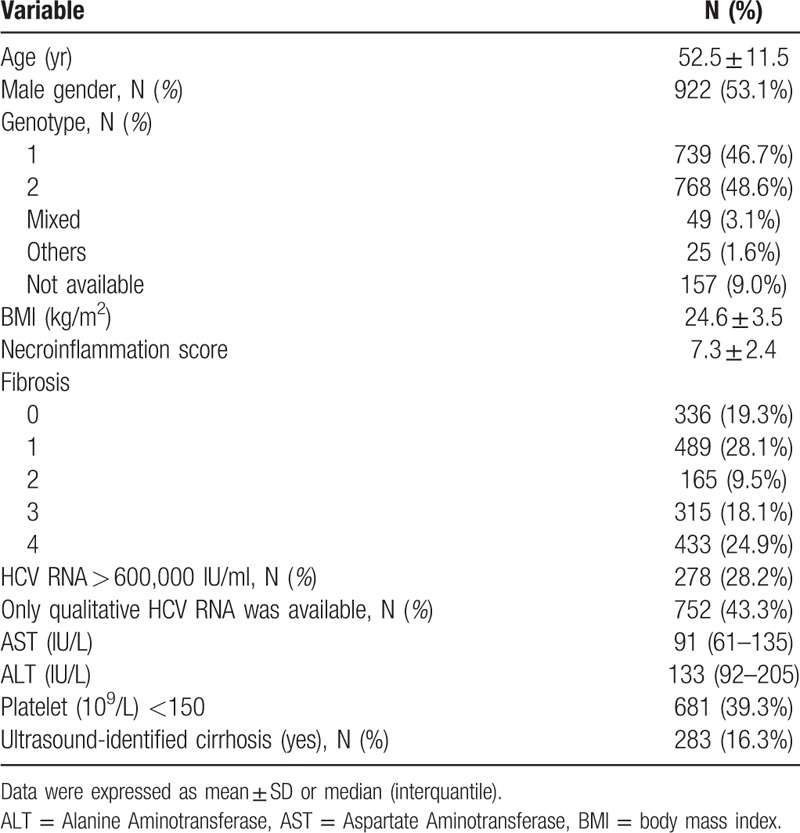
Characteristic of patients, N = 1738.

### 3.2 Comparison of patients with or without compensated cirrhosis

A comparison of the patients with or without compensated cirrhosis is shown in Table 2. In comparison to those without compensated cirrhosis, those with compensated cirrhosis were older, had higher body mass index (BMI) scores, had higher necroinflammation scores, had higher AST and ALT levels, and included higher proportions of patients who were female, genotype 1, had thrombocytopenia, and had ultrasound-identified cirrhosis.

**Table 2:**
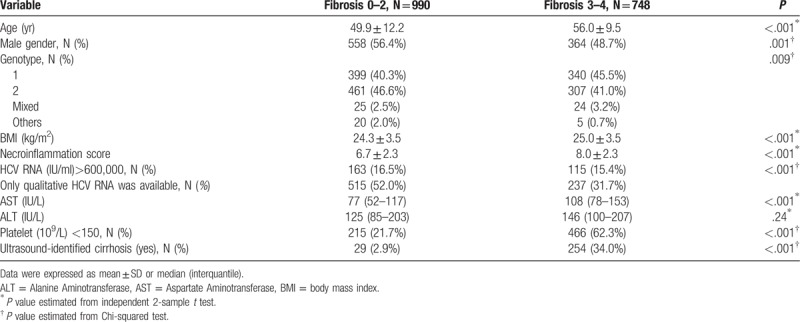
Comparison of patients with or without compensated cirrhosis (i.e., modified Knodell fibrosis score 3 or 4).

### 3.3 Diagnostic accuracy of ultrasound in identifying compensated cirrhosis

Of the 748 patients with compensated cirrhosis, 494 (66%) were negative for ultrasound-identified cirrhosis (i.e., the ultrasound yielded false-negative results); on the other hand, 29 (2.9%) of the 990 patients without compensated cirrhosis were positive for ultrasound-identified cirrhosis (i.e., the ultrasound yielded false-positive results). Using ultrasound-identified cirrhosis to diagnose compensated cirrhosis, the sensitivity was 34.0%, the specificity was 97.1%, the PPV was 89.8%, the NPV was 66.1%, the positive likelihood ratio was 11.6, the negative likelihood ratio was 0.68, and the AUROC was 0.66.

### 3.4 Ultrasound signs of portal hypertension

Ultrasound-identified cirrhosis was noted in 283 patients in our study, and splenomegaly was noted in 106 patients (37.5%).

## 4 Discussion

In this study, ultrasound was found to be highly specific in diagnosing compensated cirrhosis, with a specificity of 97.1%. However, the sensitivity was low (34.0%). Therefore, the ultrasound findings were sufficiently specific to allow a diagnostic confirmatory strategy,^[10]^ indicating that a positive result can “rule in” compensated cirrhosis; on the contrary, the sensitivity of ultrasound was too low to support a screening diagnostic strategy, indicating that a negative result cannot rule out compensated cirrhosis.^[10]^

Previous prospective studies used ultrasound-identified liver surface nodularity as a predictor of compensated cirrhosis in CHC patients, with low sensitivity (53% and 73%, respectively) and high specificity (91% and 90%, respectively), results which are compatible to those for this study.^[11,12]^

It is widely accepted that nodular liver surface can be used to diagnose cirrhosis.^[13]^ A recent study reported that mammillated caudate lobe, gallbladder scalloping, and inferior vena cava scalloping are three novel signs that improve the accuracy of ultrasound in diagnosing liver cirrhosis.^[14]^ Remarkable advances have been made in cardiovascular medical image.^[15–17]^

According to the American Association for the Study of Liver Diseases (AASLD) guideline,^[18]^ splenomegaly taken alone is a sensitive, but nonspecific, sign of portal hypertension. The presence of portocollateral vessels or a reversal of flow within the portal system is 100% specific for clinically significant portal hypertension (CSPH).^[19]^ Several sonographic signs of portal hypertension have been described, such as the reduction of portal vein velocity and dilatation of portal vein.^[20,21]^ Ultrasound-identified cirrhosis was noted in 283 patients in our study, and splenomegaly was noted in 106 patients (37.5%). Therefore, 106 patients (37.5%) had possible portal hypertension in this study. However, we did not routinely record sonographic signs of portal hypertension regarding portal vein and portocollateral vessels. In this study, only ultrasound signs for assessing hepatic parenchyma were used, whereas signs of CSPH were not included. According to the AASLD guideline recommendation, patients with compensated cirrhosis should be substaged into those with mild portal hypertension and those with CSPH.^[18]^ The presence of portocollateral vessels or a reversal of flow within the portal system are 100% specific (pathognomonic) signs of portal hypertension, such that liver cirrhosis can be diagnosed without liver biopsy with these signs. ^[19]^ However, using this non-invasive criteria to diagnose liver cirrhosis could have led to the under diagnosis of liver cirrhosis in those without CSPH.

Distinguishing between a stage of fibrosis 3 or 4 is also important for choosing type and duration of DAAs. According to the EASL guideline, ^[3]^ patients with cirrhosis (F4) must be identified, as their treatment regimen must be adjusted. Three kinds of ultrasound-based elastography for prediction of F3 (bridging fibrosis) and F4 (cirrhosis) were mentioned in the EASL guideline. ^[3]^ These ultrasound-based elastography including TE, ^[22–24]^ acoustic radiation force impulse imaging (ARFI)^[25]^ and 2D-shear wave elastography (2D-SWE).^[26]^ Overall diagnostic performances of these ultrasound-based elastography were excellent with the AUROC > 0.90 for prediction of F4.^[22–26]^ In our study, ultrasound is highly specific in diagnosing compensated cirrhosis (≥F3) in CHC patients. However, the sensitivity is low (34.0%) The sensitivity of these ultrasound-based elastography in diagnosing compensated cirrhosis (≥F3) is high (range from 72∼90%).^[22,25,26]^

Theses ultrasound-based elastography techniques have been fully described.^[27]^ According to the EASL guideline,^[4]^ TE have several advantages:

1. most widely used technique,
2. good reproducibility,
3. good performance for cirrhosis, and
4. easy to learn, can be performed by a technician after minimal training.

ARFI have several advantages:

1. It can be implemented on a regular ultrasound machine,
2. higher applicability than TE (e.g., obesity and ascites),
3. the location of region of interest (ROI) can be chosen by the operator, and
4. good performance for cirrhosis.

2D-SWE has several advantages:

1. It can be implemented on a regular ultrasound machine,
2. the location of ROI can be chosen by the operator,
3. good performance for cirrhosis, and
4. good applicability.

The major strength of this study is that it included a large cohort of treatment-naïve patients, because several studies have shown that liver histology may improve even among nonresponders to interferon-based therapy.^[28–30]^ Most importantly, we used ultrasound to evaluate hepatic parenchyma alone. The use of this simple technique in our study is not time consuming and more feasible for daily clinical practice. We acknowledge, however, that there are limitations to this study. First, it was a retrospective study. Second, it included patients from a medical center, 43% of whom had compensated cirrhosis on histology and none of whom had prior antiviral treatment. As such, whether our results can be generalized to community-based practices in which patients may have milder disease or to patients who have failed prior interferon therapy remains to be determined. Third, our study did not involve a central pathologist for the interpretation of liver histology. There were interobserver discrepancies in the assessments of hepatic fibrosis.

In conclusion, ultrasound is insensitive but highly specific for the detection of compensated cirrhosis in CHC patients in real world clinical practice. Assessment of the compensated cirrhosis by ultrasound-based elastography is not required in CHC patients with ultrasound-identified cirrhosis. The sensitivity of ultrasound-based elastography in diagnosing compensated cirrhosis is high.^[22,25,26]^ Thus, we suggest ultrasound-based elastography in CHC patients without ultrasound-identified cirrhosis.

## Author contributions

**Conceptualization:** Yi-Hao Yen.

**Data curation:** Yi-Hao Yen.

**Formal analysis:** Yi-Hao Yen.

**Funding acquisition:** Yi-Hao Yen.

**Investigation:** Yi-Hao Yen.

**Methodology:** Yi-Hao Yen.

**Project administration:** Yi-Hao Yen.

**Resources:** Yi-Hao Yen.

**Supervision:** Fang-Ying Kuo, Chien-Hung Chen, Tsung-Hui Hu, Sheng-Nan Lu, Jing-Houng Wang, Chao-Hung Hung.

**Writing – original draft:** Yi-Hao Yen.

Tsung-Hui Hu orcid: 0000-0002-9172-1967.
